# Comparison of Two Different Positions for Ultrasound-Guided Intervertebral Distance Evaluation

**DOI:** 10.4274/TJAR.2023.231277

**Published:** 2023-12-27

**Authors:** Feyza Aksu, Ferda Kartufan, Özge Köner, Ayşegül Görmez, Elif Çiğdem Keleş

**Affiliations:** 1 Yeditepe University Faculty of Medicine, Department of Anaesthesiology and Intensive Care, İstanbul, Turkey; 2 İstinye University Faculty of Medicine, Department of Anaesthesiology, İstanbul, Turkey; 3 Yeditepe University Faculty of Medicine, Department of Radiology, İstanbul, Turkey; 4 Yeditepe University Faculty of Medicine, Department of Biostatistics, İstanbul, Turkey

**Keywords:** Anatomy, lumbar intervertebral distance, neuraxial anaesthesia, patient position, ultrasonography

## Abstract

**Objective:**

During neuraxial anaesthesia, correct patient positioning is key for increased block success and (patient) comfort. The aim of this prospective study was to compare the lateral fetal decubitus (LFD) position with the sitting fetal lotus (SFL) regarding interspinous distance, transverse diameters of paravertebral muscles measured with ultrasonography, and patient comfort.

**Methods:**

Fifty adult participants who could sit cross-legged and had no lumbar anomalies were included in our prospective study. In both SFL and LFD positions, measurements were performed with ultrasonography; in the axial plane, interspinous distance at the level of L4-L5, in the sagittal plan, with the probe slightly tilted, subcutaneous tissue-spinous process depth, and transverse diameters of paravertebral muscles were measured. Stretcher, waist position, and abdominal comfort were scored on a scale of 1 (very bad) to 7 (perfect) with a verbal numeric satisfaction scale.

**Results:**

Interspinous distance was significantly larger in the SFL position than in the LFD position (*P* < 0.05). There was no significant difference between the two positions (*P* > 0.05) regarding patient comfort. Paravertebral muscle diameters were significantly broader in the SFL position than in the LFD position. The diameter of the left paravertebral muscle in the SFL position (45.8±8.8 mm) was larger than that in the LFD position (43±7.8 mm; *P* < 0.001). The diameter of the right paravertebral muscle in the SFL position was (47±9 mm) larger than that in the LFD position (43.4±7.6 mm; *P* < 0.001).

**Conclusion:**

Although there was no difference regarding the comfort between the two positions, the interspinous distance was larger in the SFL position than in the LFD position.

**Main Points**

• Interspinous distance measured with ultrasonography at the lumbar 4th and 5th vertebral level has been shown to be significantly wider in the sitting fetal lotus position than in the lateral fetal decubitus position.

• There was no difference between the two positions in terms of patient comfort.

• Paravertebral muscle width is more relaxed in the sitting fetal lotus position than in the lateral fetal decubitus position.

## Introduction

Although neuraxial anaesthesia techniques have been shown to be highly reliable, failed and inadequate blockage is worrying for both anaesthesiologists and patients and can turn the advantages of regional anaesthesia into disadvantages.^1^ The failure rate of neuraxial block is around 2-20%.^2^ Identifying the preventable causes of failure is critical for the accuracy of implementation and patient safety. Body structure, spinal anatomy, unidentified anatomical landmarks, excess body weight (body mass index ≥30 kg m^2^), inappropriate patient position, intervention method, experience of the anaesthesiologist, and equipment are considered as the reasons for failure.^1^

Ultrasonography (USG) was introduced into clinical practice in the 90s to guide neuraxial block before or during the procedure.^3^ Following the development of technology, USG image quality has improved, and its use during neuroaxial block has gained popularity. New USG devices provide better visualization of the bone structure surrounding the spinal canal. A meta-analysis has proven the increased intervention success of neuraxial anaesthesia performed using USG.^4^

Correct position, comfort of the patient, and sufficient interspinous space are essential for successful neuraxial implementation; therefore, different positions such as sitting fetal position, lateral decubitus fetus position, sitting fetal holding ball on lap position, sitting fetal semi-calf flexion position, 30% angle table position, modified sitting position, Oxford position, and cross-leg position have been described.^5,6,7,8,9^

In our study, we compared the lateral fetal decubitus (LFD) position with “sitting fetal lotus (SFL) position” in terms of interspinous distance (ISD) and depth of anatomical structures measured at L4-5 intervertebral space by USG and in terms of patient comfort. We hypothesized that the SFL position is superior to the frequently used LFD position in terms of interspinous distance and patient comfort.

## Methods

This study was approved by the Yeditepe University Clinical Research Ethics Committee (date: 02.01.2019, approval no: KAEK: 923). The research was recorded with the ID number NCT03889223 at ClinicalTrails.gov protocol registration and results system-PRS U.S. National Library of Medicine Ultrasonographic assessments and satisfaction surveys for 50 volunteers were conducted after informed consent was obtained between March 20, 2019 and June 25, 2019.

Fifty healthy adult volunteers (older than 18 years-old) who had no lumbar anomaly, had not undergone lumbar regional surgery, and could sit cross-legged were included in our study. Demographic data of the participants, such as gender, age, height, body weight, and body mass index, were recorded. Volunteers were evaluated in the same room by the same radiologist using the same USG device [General Electric LOGIQ E9 (CISPR11 Group 1 Class A), Wauwatosa, WI, USA, 53226) and probe (9L-linear, probe 2.4-10 MHz)].

**Figure 1:**
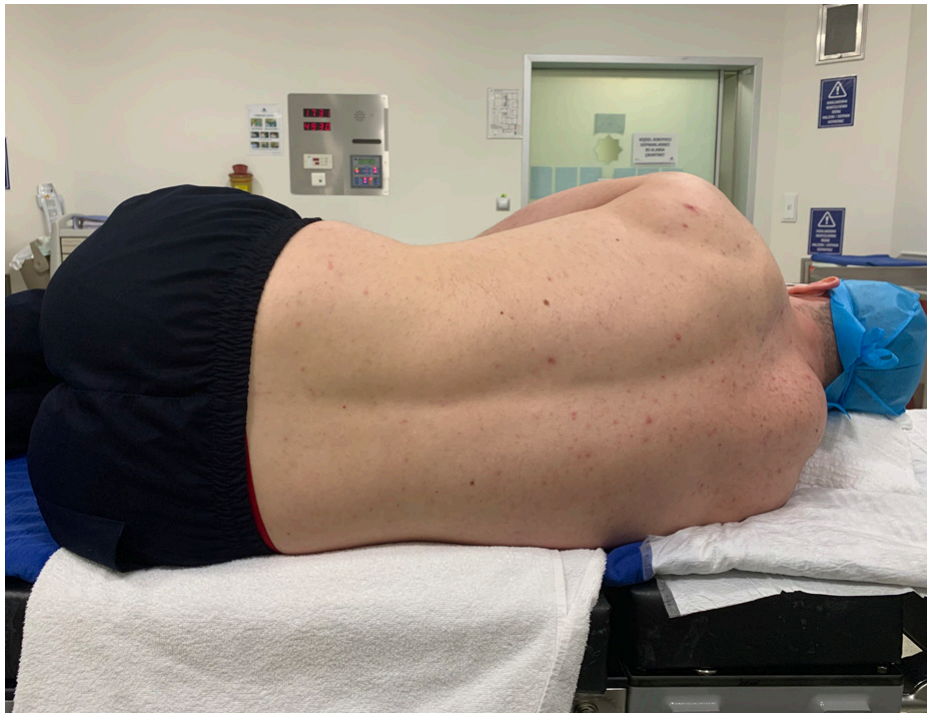
Lateral fetal decubitus position

Participants were first placed in the LFD position. In the LFD position, participants were laid in the left lateral decubitus position, with the back toward the radiologist, their chins leaned to their chest, and their knees and hips were flexed thoroughly (Figure 1). The right and left crista iliaca were palpated, and the line connecting the posterior superior iliac wings of both crista iliaca in the horizontal plane was determined as the Tuffier line. Measurements with ultrasound were performed at the L4-L5 intervertebral space on the Tuffier line. After applying the hydrophilic anti-allergic USG gel, the area from the sacrum to the Tuffier line was examined in the longitudinal sagittal plane. The thickness of subcutaneous tissue (ST), skin to spinous process (S-SP) depth, and transverse diameters of the right and left paravertebral muscles were measured with USG in the axial plane at the level of the L4-L5 interspinous space. The interspinous distance was measured at the L4-L5 level in the same position.

Afterwards, the participants were placed in a SFL position with their legs crossed, their back turned toward the radiologist, their chin leaned to the chest and arms rested on the knees, and then asked to hunch their back (Figures 2a, 2b). As with the LFD position, the Tuffier line is identified in the SFL position as well. The area from the sacrum to the Tuffier line was examined in the longitudinal sagittal plan. The transverse diameters of the bilateral paravertebral muscles, interspinous distance at the L4–L5 level, thickness of subcutaneous tissue, and depth from the subcutaneous tissue to spinous processes were measured in the axial plane with the probe slightly inclined. The interspinous distance was also measured at the L4-L5 level in the same position. USG measurements were recorded in millimeters. Ultrasonographic images of the lumber region are shown in Figures 3a and 3b.

After the USG evaluation was completed, participants were asked to rate their position experience using a 7-point Likert numerical comfort assessment questionnaire.^10^ The stretcher comfort, position comfort, waist comfort, and abdominal comfort were scored as 1 (very bad) - 7 (perfect) for both positions.

### Statistical Analysis

Eighteen participants were required to confirm a 20% change in interspinous difference based on a preliminary evaluation between the groups (1-β=0.9; alpha=0.05). However, at least 48 participants were required to compare the comfort with a 7-point Likert numerical assessment questionnaire. Neuraxial anaesthesia is applied to approximately 600 patients per year in our hospital, and the number of samples required to reflect this population was found to be a minimum of 235 with the corresponding table of reliability level of 95%. The frequency range is accepted as 3 and from the 150-person volunteer pool, 50 people were chosen as being the 1^st^, 4^th^, 7^th^, 10^th^...^11^ Volunteers were given a code, and informed consent was obtained. The data are reported as the mean (standard deviation) and minimum-maximum. The distribution of variables was evaluated using the coefficient of variation, skewness-kurtosis, normality test of Shapiro-Wilk, and histogram. Parametric tests were used for the analysis of data with normal distribution. Student’s t-test and Wilcoxon test were used in dependent samples to compare the USG data of the two positions. The t-test and Mann-Whitney U test were used in independent samples to compare the sonographic results of the two positions by gender. In addition, the Mann-Whitney U test was used to compare differences in USG measurements from a gender perspective. The marginal homogeneity test was used to evaluate the 7-point comfort survey, and the chi-square test (Fisher’s Exact test applied as being Monte Carlo confidence level 95%) was used to analyze the comfort change in relation to gender-based positioning techniques. IBM statistic packages for the social sciences 22.0 program (IBM SPSS Corp; Armonk, USA) were used for analysis. A “*P*” value less than 0.05 was considered statistically significant.

## Results

Demographic data of 50 volunteers are presented in Table 1.

The average ST and S-SP measured in the SFL position were significantly shorter than those measured in the LFD position. The subcutaneous tissue thickness was 8.8±5 mm in the SFL position, whereas 9.8±5.2 mm in the LFD position (*P* < 0.001). The skin to spinous process distance was 11±5.2 mm in the SFL position and 12±5.5 mm in the LFD position (*P* < 0.001; Table 2).

The left and right paravertebral muscle diameters were significantly broader in the SFL position than in the LFD position. The diameter of the left paravertebral muscle in the SFL position (45.80±8.82 mm) was larger than that in the LFD position (43.04±7.68 mm; *P* < 0.001). The diameter of the right paravertebral muscle in the SFL position was (47±9 mm) larger than that in the LFD position (43.4±7.6 mm; *P* < 0.001). The diameter of the mean paravertebral muscle was broader in the SFL position (46.5±9) than in the LFD position (43±7.6; *P* < 0.001) as well. The interspinous distance was significantly larger (17.5±2 mm) in the SFL than in the LFD (14.7±2 mm; *P* < 0.001) position (Table 2).

When differences between LFD and SFL positions were compared according to gender, no significant difference was observed in terms of ST (*P*=0.092), S-SP (*P*=0.271), mean paravertebral muscle (*P*=0.080), and interspinous distance (*P*=0.694; Table 3).

According to the seven-point Likert comfort evaluation scale, there was no significant difference between the two positions in terms of stretcher comfort (5.9±1.3 vs 5.8±1.3, in LFD and SFL positions; respectively, *P*=0.599), position comfort (5.2±1.6 vs 5.4±1.4, in LFD and SFL positions; respectively, *P*=0.490), abdomen comfort (5±1.6 vs 5.5±1.3, in LFD and SFL positions; respectively, *P*=0.135), and lumbar comfort (5.3±1.5 vs 5.4±1.3, in LFD and SFL positions; respectively, *P*=0.631) (Table 4).

## Discussion

The SFL position is advantageous regarding USG-based measurements compared to the LFD position. The interspinous distance is significantly wider in the SFL position than in the LFD position. There was no significant difference between the two positions in terms of patient comfort.

Neuroaxial anaesthesia is performed in three main positions (sitting position, lateral decubitus position, prone position). However, other modified positions (modified sitting position, mid-calf position, holding the ball on the lap position, angled table position, Oxford position, cross-leg position) have also been described. As far as we know, there are only two studies in which patients were placed in the sitting lotus position. In one of the aforementioned studies, patients were in the sitting lotus position and holding a pillow on their lap.^6,9^ In a previous study performed by us, patients were placed in the SFL position; however, patients’ arms were rested on their knees.^12^

There was no difference in spinal anaesthesia success between frequently used lateral decubitus fetal and conventional sitting positions.^13^ In their study, Manggala et al.^8^ could not find a difference between the crossed-leg sitting position, which resembles our SFL position the most, and the conventional sitting position in terms of spinal anaesthesia success. In the aforementioned study, the comfort of the position was not evaluated. In our study, all the participants were given both positions consequently. Therefore, they were able to compare the comfort of both positions.

Successful neuroaxial anaesthesia intervention can be achieved with an adequate interspinous distance and appropriate patient position.^14^ Positioning the patient properly and maintaining the position by keeping the patient comfortable will help the ISD to remain unchanged, thus increasing the chance of success of neuroaxial anaesthesia.^14^

Meta-analysis^4,15^ has shown that the use of USG significantly improves the success and effectiveness of neuroaxial anaesthesia. Besides USG, there are other imaging methods such as magnetic resonance imaging, fluoroscopy, and computed tomography to measure interspinous distance and other surrounding tissues, but USG is noninvasive and easily accessible.

The reliability of USG is associated with the clinical experience of the researcher. USG can better demonstrate anatomical signs and measurements of the anatomy of the spine in the hands of a skilled and experienced specialist, even if the patient is obese and pregnant.^16,17^ Therefore, an expert radiologist performed the evaluation using USG.

The only study in the literature comparing the comfort of neuroaxial positions is by Dimaculangan et al.^6^ In their study, the authors compared six different positions for ISD with sonography and comfort with a 10-point VAS score and found interspinous distance wider in the “sitting fetal position” than in other sitting positions. In our study, the interspinous distance measured at the level of L4-5 vertebrae was significantly wider in the SFL position than in the LFD position. Furthermore, the sitting fetal position is more comfortable than the conventional sitting position.^6^ In the authors’ study^6^ the sitting fetal position was different from our SFL position; the subjects sat on the side of an OR table, thighs on the table with legs hanging freely over the table’s edge, arms resting on their legs with the back curved. In their study, Dimaculangan et al.^6^ found the sitting fetal position, with legs hanging freely over the table’s age, as the 3^rd^ most comfortable position after the sitting position holding a ball on the lap and lateral decubitus position.

In our study, ST and S-SP measurements were also significantly shorter in favor of the SFL position. In a previous study, we showed that enlarged paraspinal muscle diameter was correlated with increased patient comfort.^12^ To the best of our knowledge, no other studies have shown the correlation between paraspinal muscle relaxation and patient comfort before our aforementioned study. Further relaxation of the paraspinal muscles in the SFL position may help reduce pain in injection interventions during epidural and spinal anaesthesia; therefore, patient comfort might be better during the procedure. This can both increase patient compatibility and facilitate the procedure by providing better stabilization. Consequently, it can help perform a more successful neuroaxial block. In our current study, transverse diameters of paraspinal muscles measured using USG revealed a significant increase in favor of the SFL position. However, this finding has not yielded better comfort in favor of the SFL position. In our study, a 7-point Likert comfort score was used to compare the volunteers’ comfort in the LFD and SFL positions. The SFL position was superior to the LFD position in all parameters measured by USG. However, there was no statistical difference between the two positions regarding comfort. An explanation for this situation might be that the participants feared falling from the stretcher because they were seated parallel to the long edge and in the middle of the stretcher. However, patients sat cross-legged perpendicular to the short axis of the OR table in the SFL position; therefore, there was a perceived or actual risk of falling.

There were no significant differences between the genders in terms of USG measurements. Therefore, the SFL position can be used in both genders. Shorter ST and S-SP distances provided with the SFL position suggest that this position may be beneficial in obese and pregnant patients compared with the LFD position.

### Study Limitations

This study has some limitations. The SFL position was not compared with other traditional positioning techniques in terms of neuroaxial block success rate. In this study, morbidly obese and elderly patients were not included. However, no other studies have compared paraspinal muscle measurements with USG and evaluated patient comfort using the 7-point Likert comfort scale.

## Conclusion

Although no difference was found in terms of patient comfort between the two positions, SFL is advantageous in USG measurements compared with the LFD position. The interspinous distance is significantly wider in the SFL position than in the LFD position. Despite not being evaluated in this study, it may be suggested that the SFL position may increase the success of neuroaxial block. Considering these findings, we believe that the future studies should evaluate the reliability and success of the SFL position during neuroaxial block.

## Figures and Tables

**Table 1:**
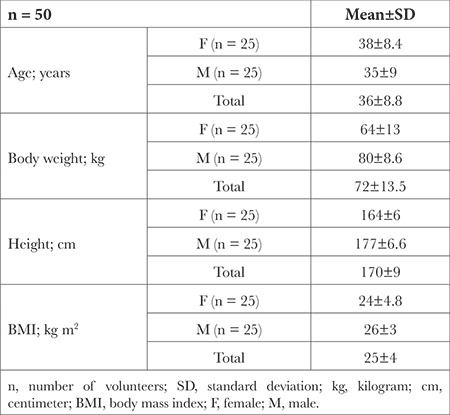
Demographic Data of Volunteers

**Table 2:**
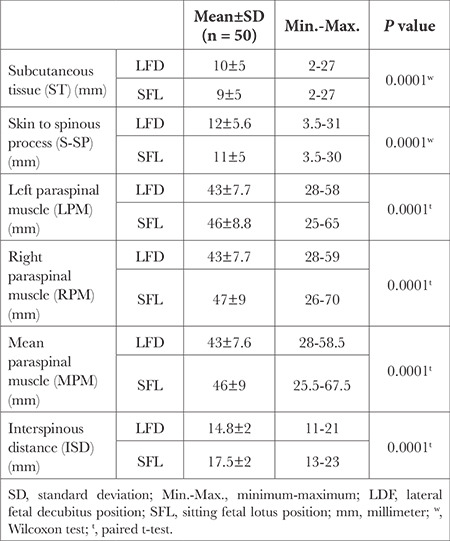
Ultrasonography Measurements of the Volunteers

**Table 3:**
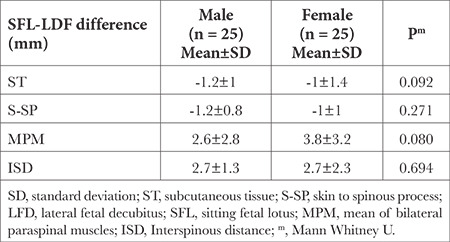
Ultrasonography Measurements of Difference Between SFL and LFD Positions According to Gender

**Table 4:**
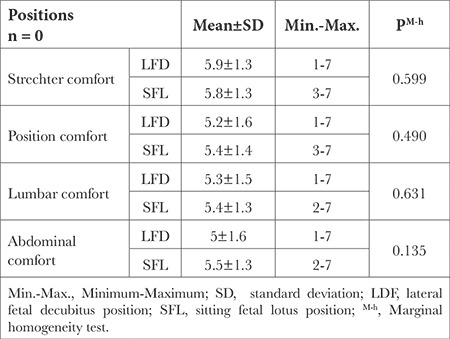
7-point Likert Comfort Score Comparison

**Figure 2a:**
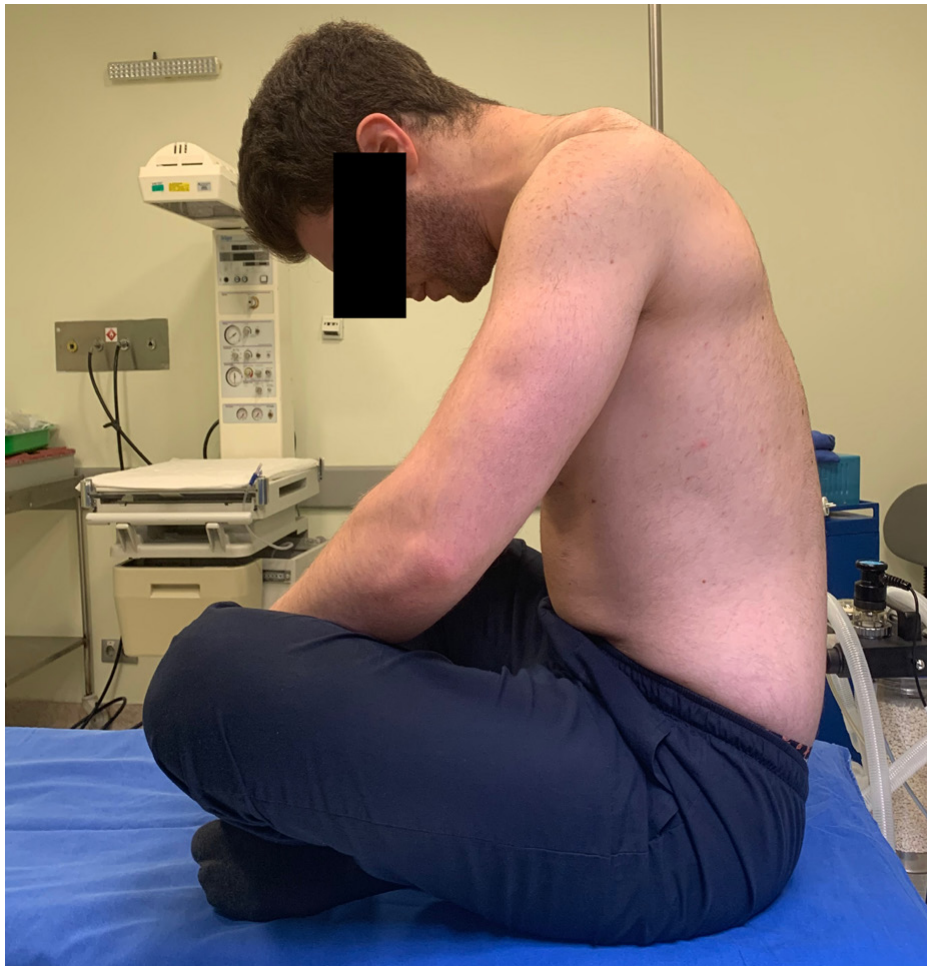
Sitting fetal lotus position, lateral view.

**Figure 2b:**
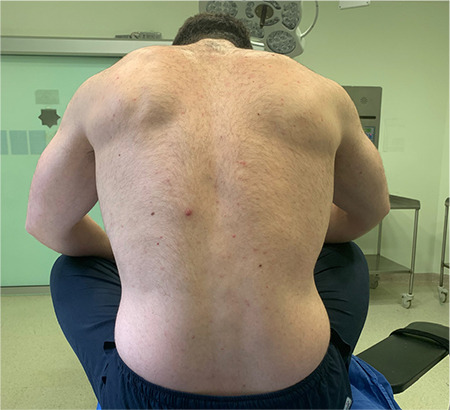
Sitting fetal lotus position, posterior view.

**Figure 3a:**
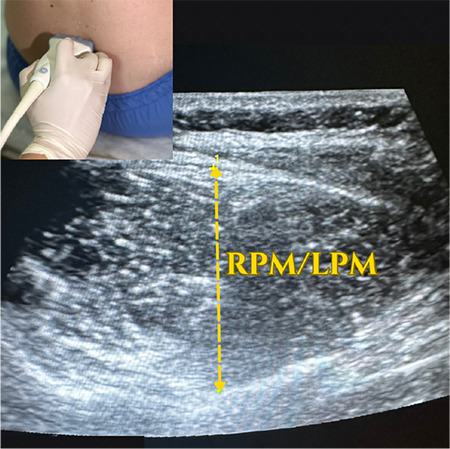
USG image of examined lumber vertebral region. USG, ultrasonography; RPM, right paraspinal muscle; LPM, left paraspinal muscle.

**Figure 3b:**
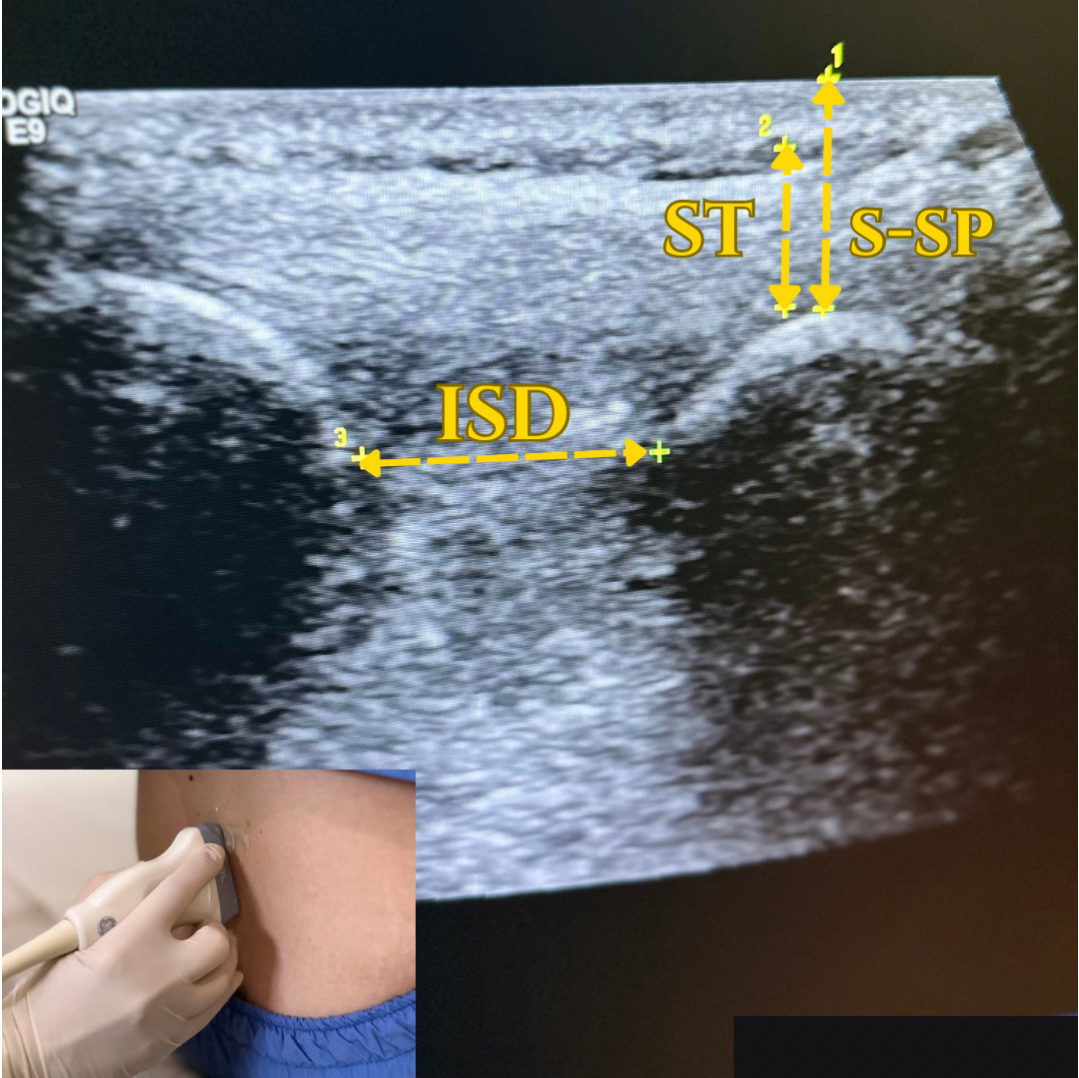
Enlarged lumber vertebral USG view. USG, ultrasonography; ISD, interspinous distance; ST, subcutaneous tissue; S-SP, skin to spinous process.
